# Veto player theory and reform making in Western Europe

**DOI:** 10.1111/1475-6765.12226

**Published:** 2017-07-26

**Authors:** MARIYANA ANGELOVA, HANNA BÄCK, WOLFGANG C. MÜLLER, DANIEL STROBL

**Affiliations:** ^1^ University of Vienna Austria; ^2^ Lund University Sweden

**Keywords:** veto players, reform making, policy output, Western Europe

## Abstract

Veto player theory generates predictions about governments’ capacity for policy change. Due to the difficulty of identifying significant laws needed to change the policy status quo, evidence about governments’ ability to change policy has been mostly provided for a limited number of reforms and single‐country studies. To evaluate the predictive power of veto player theory for policy making across time, policy areas and countries, a dataset was gathered that incorporates about 5,600 important government reform measures in the areas of social, labour, economic and taxation policy undertaken in 13 Western European countries from the mid‐1980s until the mid‐2000s. Veto player theory is applied in a combined model with other central theoretical expectations on policy change derived from political economy (crisis‐driven policy change) and partisan theory (ideology‐driven policy change). Robust support is found that governments introduce more reform measures when economic conditions are poor and when the government is positioned further away from the policy status quo. No empirical support is found for predictions of veto player theory in its pure form, where no differentiation between government types is made. However, the findings provide support for the veto player theory in the special case of minimal winning cabinets, where the support of all government parties is sufficient (in contrast to minority cabinets) and necessary (in contrast to oversized cabinets) for policy change. In particular, it is found that in minimal winning cabinets the ideological distance between the extreme government parties significantly decreases the government's ability to introduce reforms. These findings improve our understanding of reform making in parliamentary democracies and highlight important issues and open questions for future applications and tests of the veto player theory.

## Introduction

Governments deal on a daily basis with challenges of the ongoing financial crisis, refugee influx, economic globalisation, aging society and resulting problems related to the social and economic welfare of the country and its citizens. In situations when policy change is necessary, it is desirable that political systems are responsive, able to adapt to new conditions and to solve problems when they arise. Some governments can introduce reforms smoothly and can rapidly adapt their policies to changes in external circumstances, others, however, delay or fail to adopt reforms even when policy inaction leads to high societal costs.

There are different central explanations for policy change. Tsebelis's (1995, 1999, 2002) veto player theory provides probably the most general political explanation for policy stability and change across different regime types, party systems and legislative types, accounting for the political actors’ policy preferences and institutional constraints. Veto player theory predicts that, *ceteris paribus*, policy stability will be the result of a greater number of veto players with greater ideological differences between them. As a theory of legislative decision making, it makes predictions about the amount of significant laws that change the policy status quo. However, because of the difficulty in separating significant from incremental legislation, there are only a few studies explicitly focusing on law production, which are either single‐country studies or cover only a limited number of policy reform areas (Kreppel 1997; Tsebelis 1999, 2002; Conley & Bekafigo 2010). Instead, scholars interested in the role of veto players in policy making typically investigate patterns of spending, taxation and budgetary changes.

We address the veto player theory by analysing the reform productivity of governments across countries and time and evaluate its predictive power in a combined model with other – economic and political – explanations for policy change (Clarke & Primo 2012; Ganghof 2017). For this purpose, we have compiled a dataset on about 5,600 important government reform measures in the areas of social, labour, economic and taxation policy undertaken in 13 Western European countries during 20 years from the mid‐1980s until the mid‐2000s. We have extracted significant government reform measures by manually coding more than 1,000 periodical country reports of the Economist Intelligence Unit (EIU). The reform‐specific information in these reports is prepared by country experts aiming to inform business circles, international organisations and government agencies about policy changes in a given country. In addition, we have cross‐validated and extended this coding of reform measures from EIU reports using the periodical Organisation for Economic Cooperation and Development (OECD) Economic Surveys.

We seek to analyse reform productivity by taking into account different economic, political and institutional factors. Political economists expect that governments respond to the current economic conditions in a country and produce more socioeconomic reforms when the country faces economic recession. In contrast, partisan theory and more recent work on coalition policy making, consider partisan preferences as the main driving force behind policy change. Veto player theory, in turn, highlights the central role of veto powers of the political actors involved in the decision‐making process for policy change and policy stability (Tsebelis 1999, 2002). Here policy change is expected when the ‘winset’ – the area with policy alternatives which makes all veto players better off – is nonempty, or the size of the core – the area of policy alternatives which make at least one veto player worse off – is small.

We find that economic demand and partisan policy demand are highly important for policy change and governmental reform activity. In particular, our analysis reveals that policy demands due to macroeconomic crisis events and greater ideological alternation between governments result in more reforms. We do not find empirical support for the veto player theory in its pure version, which predicts that governments’ ability to change the policy status quo decreases with greater ideological distance between the veto players in all types of governments. Following insights from the work by Strøm (2000), who questions the veto powers of surplus parties in oversized coalitions, and Ganghof and Bräuninger (2006), Ganghof (2006) and Curini and Zucchini (2011), who point out several reasons why opposition parties should be considered as potential veto players in the decision‐making process of minority governments, we introduce government type as a variable. We find support for the predictions of the veto player theory for the special case of minimal winning cabinets, but not for minority and oversized governments. Our findings have important implications for future applications and testing of the veto player theory, and for our understanding of reform making in parliamentary democracies.

## Veto players and policy change: An overview

Understanding the determinants of policy change has been a longstanding interest among a variety of social science disciplines. Among political economists, economic factors such as globalisation and market conditions have been researched as determinants for government reforms. From a political economy perspective, reforms are expected in times of economic crises. Here the existing literature argues that governments adapt the policy status quo when economic circumstances become sufficiently poor (e.g., Drazen & Easterly 2001). From a political perspective, however, policy change is driven by the preferences of the involved political actors, who strive to implement their policy interests (Hibbs 1977), and are expected to invoke policy change when they face a policy status quo that conflicts with their ideological position.

Tsebelis's (1999, 2002) veto player theory assumes policy‐seeking actors strive to change the policy status quo, and takes into account the political‐institutional context which constrains them to do so. Tsebelis combines the importance of policy preferences of political actors with the institutional constraints they face in a parsimonious theory, offering an explanation for policy stability across different political regime types. Greater policy stability is expected in political systems with a greater number of, and greater ideological distance between veto players – the actors whose approval is required for a policy change.

The seminal studies on the veto player theory by Tsebelis (1995, 1999, 2002) have triggered a wide‐ranging interest. Analyses applying, testing and extending the predictions of the veto player theory continue to mushroom in the field. Veto player theory has proven to be exceptionally fertile, allowing for a wide range of observable implications across political systems and for various types of data. Existing studies test predictions of the theory on policy change, predominantly relying on macroeconomic outcome data, such as budget allocations, budget deficits, inflation rates, spending and taxation (for an overview, see Hallerberg 2011). Among many other applications of veto player theory, scholars study the effect of veto players on cabinet stability and duration (Saalfeld 2011), cabinet formation (Tsebelis & Ha 2014; Eppner & Ganghof 2017), discretion of central banks (Bernhard 1998; Keefer & Stasavage 2003), economic growth (MacIntyre 2001), referendums (Hug & Tsebelis 2002), voter turnout (Carlin & Love 2013), bureaucratic corruption (Bagashka 2014), as well as human rights (Lupu 2015).

Many existing studies provide support for the predictions of the veto player theory. For example, in a study on budget decisions of 19 OECD countries for the period 1973–1990, Tsebelis and Chang (2004) show that greater ideological distance between veto players leads to fewer budgetary changes. Using data on 19 OECD countries for a roughly 30‐year period, Bräuninger (2005) finds that veto players have also a substantive impact on changes in government expenditure. With respect to government deficit and inflation, scholars have shown that countries with many veto players tend to maintain the status quo, lock themselves into the same pattern of either high or low budget deficit and maintain previous levels of inflation (Treisman 2000; Franzese 2002). Other studies show that predictions derived from veto player theory can explain government spending (Bawn 1999; König & Troeger 2005; Citi 2015), taxation (Hallerberg & Basinger 1998; Basinger & Hallerberg 2004; Ganghof 2006), investment decisions and economic growth (Henisz 2000; MacIntyre 2001), as well as government's ability to respond to crisis situations (Leblang & Satyanath 2006).

Although veto player theory makes predictions about policy change, which are induced through legislative instruments, only a handful of studies focus directly on legislative output and the ability of governments to introduce reforms. In an analysis of all bills initiated in Italy between the 1950s and the 1990s, Kreppel (1997) finds that legislative output decreases with a higher number of parties in government. However, the analysis does not explicitly differentiate between significant and incremental legislation and does not investigate the impact of ideological range between government parties. Including incremental laws may result in biased results as such laws may not induce changes of the policy status quo.

Due to the difficulty of differentiating between incremental and significant reforms, studies on veto player theory and legislative output often focus on reform making in single countries (Conley & Bekafigo 2010), single policy areas (Tsebelis 1999; Becher 2010; Immergut & Abou‐Chadi 2014), or on specific types of legislation like treaty ratifications (Boockmann 2006). The seminal work by Tsebelis (1999) covers 75 significant labour laws on working time and conditions, introduced across 15 Western European countries within the ten years of 1981–1991. Despite the small number of observations, Tsebelis shows that ideological range between the veto players has a significant effect on policy change.

Given the rather small sample size in Tsebelis (1999), scholars have stressed the need for further tests of veto player theory predictions on law production (see, e.g., Ganghof 2003). Follow‐up studies testing predictions of the veto player theory on law production report mixed results. Becher (2010) bases his analyses on a larger sample. He examines how veto players matter for labor market reforms introduced in 20 advanced industrial democracies from 1973 to 2000, and shows that veto players constrain the agenda‐setting power of ministers. In contrast, Conley and Bekafigo (2010) analyse the effect of ideological range between veto players on the number of significant bills initiated in Ireland between 1949 and 2000, but find only a marginally negative effect of ideological range. Similarly, Boockmann (2006) does not find support for the veto player theory's predictions in his analysis on the ratification of 52 conventions of the International Labour Organisation adopted in 17 industrialised democracies.

In addition to these empirical ‘setbacks’, the definition of veto players has been questioned. Tsebelis defines a veto player as an individual or collective actor whose agreement is required for policy change, and differentiates between institutional (e.g., legislative chambers, presidents, courts) and partisan veto players (i.e., parties that are members of a government coalition) (Tsebelis 1995: 301, 2002). He further clarifies that

there is one important difference between institutional and partisan veto players: according to the constitution, the agreement of institutional veto players is a necessary and sufficient condition for policy change, while the agreement of partisan veto players is, *strictly speaking*, neither necessary nor sufficient. (Tsebelis 1995: 302; emphasis in original)

Agreement of partisan veto players is not sufficient for policy change because a proposal that is approved by all government parties still requires a parliamentary majority and can be defeated in parliament. This can happen in instances when government parties have low levels of discipline and cannot prevent their own MPs from voting against the government proposal. It can also happen in minority governments, where government parties need the support of the opposition to reach a parliamentary majority. Approval of partisan veto players is not necessary for policy change because governments may include parties whose preferences can be bypassed or played off against each other (Tsebelis 1995: 303). This is the case in oversized majority governments, where at least one cabinet party is superfluous for building a parliamentary majority. Thus, strictly speaking, not all government parties in oversized cabinets should have a *de facto* veto player status. In essence, only in minimal winning majority governments are all parties sufficient and necessary for policy change, which raises the question whether veto player theory is applicable to all cabinet types.

Although there can be instances when the agreement of partisan veto players (all government parties) is neither necessary nor sufficient for policy change, Tsebelis (1995, 1999: 594, 2002: 96) advocates a position where all government parties, irrespective of government type, are veto players. In particular, he expects that, on average, all cabinet parties in oversized coalitions will nevertheless function as veto players, because ‘ignoring coalition partners, while numerically possible, imposes political costs’ (Tsebelis 1995: 304; see also Tsebelis 1999: 594, 2002: 96). Passing a bill against the will of a cabinet partner might lead to serious disagreement and consequently to a government crisis, where the small partner decides to resign. Of course, the resignation of a small surplus party might not necessarily lead to new elections, as the remaining partners would still command a parliamentary majority, but might still be politically dangerous (in particular, as like‐minded parties may follow). Indeed, Tsebelis assumes that there must be political factors necessitating oversized coalitions. And in order to keep the coalition intact the preferences of the different partners (even the surplus ones) must be respected, which renders each partner in the coalition a veto player.

With respect to minority governments, Tsebelis (1999: 594) argues that in practice all government parties in such cabinets are sufficient for policy change, because they ‘are equipped with significant positional and institutional weapons that enable them (most of the time) to impose their will on parliament, just as majority governments do’ (see also Tsebelis 2002: 98). Usually minority governments are centrally located (Laver & Schofield 1998), which allows them to lean towards one or another possible partner and get their policies approved in parliament. This means that, assuming policy‐seeking parties, a minority party, which is centrally located, does not need a formal ally to get its policies passed (Laver & Schofield 1998), and is therefore necessary and sufficient for policy change.

There are those who question these assumptions (Strøm 2000; Ganghof & Bräuninger 2006; Curini & Zucchini 2011). Strøm (2000: 280) argues that it is true that some coalition partners can credibly threaten to resign because they are pivotal and have favourable outside options. However, he questions this privilege for surplus parties in oversized cabinets. Surplus parties, especially if they have more extreme positions and are small, face slim outside options and therefore have little incentives to resign, lose their government posts and policy influence. In such circumstances a threat of resignation by a surplus party is less credible. In addition, an actual resignation is not detrimental for the cabinet's chances of survival because the remaining parties still hold a majority. In minority situations, on the other hand, Ganghof (2010: 680) points out that a government may be ‘very exclusive at the executive stage, but it must build at least minimal winning coalitions in the legislature in order to change the status quo’. It is thus not immediately obvious which parties in parliament function as veto players. Opposition parties have reasons not to support a government's position, even if they are clearly better off in terms of policy gains as compared to the status quo (see, e.g., Ganghof & Bräuninger 2006). Tsebelis (2002) assumes purely policy‐seeking actors, but parties are also vote‐ and office‐seeking (Müller & Strøm 1999). Opposition parties might refrain from supporting the ideal position of the minority government even if it is preferable to the policy status quo. In instances when government proposals are close to the ideal point of the opposition, opposition parties might refrain from supporting the government because it is difficult for them to claim credit for government proposals (Huber 1999). In instances when government proposals are preferable to the status quo but still relatively distant from the position taken by the opposition parties, opposition parties risk facing the electoral costs for supporting the government and compromising with their ideal position (Ganghof & Bräuninger 2006).

Another reason might lie in the ‘outside’ options of opposition parties. Although minority governments are most of the time located centrally in the policy space and can ‘juggle’ with parties on their left and right, they have a weaker position than majority governments as their survival depends upon the support of the opposition (see, e.g., Saalfeld 2008). If opposition parties face good ‘outside’ options and prefer new elections and a new government formation to a constellation where the centrally located government is playing the opposition parties against each other, they can use this to their advantage and impose their position on the government. In instances where minority governments fear new elections and a new government formation process leaving them in the opposition, the preferences of opposition parties will be considered to keep the government intact. Therefore, different opposition parties may function also as legislative veto players when minority governments are in power.

In light of this possibility, Curini and Zucchini (2011) modify the veto player model for minority governments by counting the opposition parties supporting the cabinet as veto players. This modification considerably improves the performance of their model. Tsebelis's (1999) own empirical results also cast doubt whether veto player theory is equally valid for all cabinet types. He finds a negative and statistically significant effect of ideological range on policy change for all government types added together. Separate models reveal that, while the direction of the effect of ideological range is negative for all government types as predicted by the theory, statistical significance remains only for minimal winning cabinets. These results leave the predictions of the veto player theory unsupported for minority and oversized governments. However, given the low variation in the number of significant laws and the small number of these cabinets in the dataset, it is left for future research to confirm or disprove these findings.

## Theoretical expectations about policy change

We set out to compare the predictive power of veto player theory with two additional explanations for policy change based on economic demands and the policy preferences of the parties in government. We thus derive two demand‐driven hypotheses of policy change before formulating the veto player hypothesis in the context of this analysis.

### Prediction drawn from the political economy literature: Crisis‐driven policy change

When explaining the volume of significant socioeconomic policy reforms introduced across countries, researchers often focus on various socioeconomic factors necessitating policy change (e.g., Drazen & Easterly 2001; Agnello et al. 2015; Wiese 2014). While governments cope with policy challenge on a daily basis, there is both high need and high demand for policy reforms in economic crisis situations, when the country is plagued by recession, or high levels of unemployment (Rodrik 1996). While some scholars have considered this expectation as part of the ‘conventional wisdom’ (Tommasi & Velasco 1996), others acknowledge the theoretical value of the argument and put it under empirical scrutiny (e.g., Drazen & Easterly 2001; Wiese 2014; Agnello et al. 2015). Drazen and Easterly (2001: 131; emphasis in original) argue that the hypothesis is empirically testable if it is explicitly stated that ‘times need to get *very* bad (and not just bad) to induce reform’. Formulated in this manner, this hypothesis has been empirically supported using various measurements of policy reforms and economic crises (see also Wiese 2014; Agnello et al. 2015). We follow this literature and expect that governments introduce more reform measures in times of recession. Our first hypothesis about policy change is thus:

*H1* (Economic Recession): In times of economic recession, governments will implement a greater number of socioeconomic reform measures.

### Prediction drawn from partisan and coalition theory: Preference‐driven policy change

In contrast to the political economy literature's emphasis on the importance of crisis for policy change, political science research emphasises the central role of policy preferences of political actors in policy making. The impact of partisan preferences on policy output finds wide empirical support (for overviews of the ‘do parties matter’ literature, see, e.g., Imbeau et al. 2001; Hartmann 2015). Partisan theory assumes that parties and politicians care about policies, votes and offices, which directly or indirectly motivates them to implement their ideological policy preferences (Hibbs 1977; Müller & Strøm 1999). This further suggests that partisan actors strive to move the policy status quo closer to their ideological positions. Therefore, drawing on this framework we can expect that policy change should occur when government parties are dissatisfied with the current policy status quo – in other words, when the current policy status quo is located further away from the position of the government.

The policy location of the government is not straightforward to specify. Rival theories of legislative policy making suggest different perspectives whose preferences ultimately prevail and as a consequence predict different locations of the final government policy output. Building on Black's (1948) median voter theorem, where the median legislative party has a strong bargaining position (Baron 1991; Laver & Schofield 1998), bargaining theory suggests that policy outcomes will be located closer to the ideal position of the median party. Laver and Shepsle (1996) emphasise the discretion of cabinet ministers in formulating and implementing policy, which suggests that the coalition party controlling the responsible minister will get its ideal position in the policy area for which it is in charge. Another perspective expects that the final policy choices reflect a compromise between the preferences of parties in the coalition (Austen‐Smith & Banks 1988; Baron & Diermeier 2001; Martin & Vanberg 2014), and the most common assumption in the empirical literature is to approximate the policy position of the government by taking the seat‐weighted average of the positions of all government parties (Powell 2000; McDonald et al. 2004). A study by Martin and Vanberg (2014) tests each of these perspectives against each other by analysing the introduction of legislative amendments on more than 1,000 bills in Denmark, Germany and the Netherlands. They show that governments amend legislative bills to accommodate for coalition compromise. Their results suggest that the final government output reflects the seat‐weighted government compromise rather than the position of the minister proposing them or the preferences of the median party in the legislature (Martin & Vanberg 2014: 1). Building on this literature, we approximate governments’ policy positions through the seat‐weighted cabinet position.

The location of the status quo in a given policy area is even more challenging to specify. It is made up of a large body of existing laws and regulations, and thus it is effectively not possible to place it on the same spatial dimension as the ideological position of a new government. Given that governments shift the policy status quo closer to their own policy position, we can relax this strict requirement and approximate the policy status quo taking the seat‐weighted position of the previous government(s) active in a period of four years. We draw on the literature on partisan theory and on coalition policy making and expect that the number of reform measures increases with larger ideological distance between the seat‐weighted position of the current and of the previous government(s). We thus propose that:

*H2* (Ideological Alternation): The greater the ideological distance between the position of the current government and the position of the policy status quo, the higher the number of expected reform measures.

### Prediction drawn from veto player theory: Veto players hinder policy change

Following the political economy and partisan theory literature described above, the demand for policy change (either crisis‐ or preference‐driven) increases and we expect to find higher reform productivity and less policy stability. However, the ability of governments to introduce reforms is constrained by the institutional settings in which they operate. The veto player approach takes this into account and presents a parsimonious unified theory for policy making which can explain policy change and policy stability across different regime types (parliamentarian and presidential systems), electoral systems (proportional and majoritarian), party systems (two‐party and multiparty systems) and government types (single‐party and multiparty governments). The veto player theory expects greater policy stability in systems with a greater number of veto players and larger ideological discrepancies among them.

Tsebelis (1999: 593) defines a veto player as ‘an individual or collective actor whose agreement is necessary for a change of the status quo’. The basic argument underlying this definition is that in order to introduce a significant change to the policy status quo, all veto players have to agree to the proposed change. Since each veto player may have differing policy preferences, agreement on policy changes will be more difficult if the number of veto players and the ideological distance between them increase (Tsebelis 1995, 1999, 2002).

Veto player theory presents two main expectations for the impact of veto players on law production in democracies: a higher number of veto players and a greater ideological distance between them should increase policy stability (Tsebelis 1999: 594). However, if a veto player has policy preferences that can be located between the policy preferences of the other veto players, then the influence of this veto player will be ‘absorbed’ (see Tsebelis 2002). In other words, the mere number of veto players is not sufficient to predict significant policy change.

Tsebelis adapts this expectation to the specific case where veto players make policy decisions in a one‐dimensional space. This adaption presents simpler expectations, which can be tested using preference data in a single dimension, such as the left–right dimension. For the one‐dimensional case, Tsebelis (1999: 595) shows that policy stability depends on the maximum ideological distance between the veto players and not their number. According to this proposition, in a one‐dimensional space, only the policy preferences of the extreme veto players are important for policy making. Veto players located between the positions of other, more extreme veto players will be absorbed and will not affect policy stability or change. Accordingly, we expect that the ideological distance between the extreme veto players should hinder policy change, and hypothesise that:

*H3* (Ideological Range): The greater the ideological range between the veto players in the political system, the lower the expected number of introduced reform measures.

Similar to the alternation hypothesis, the veto player theory predicts policy change when the status quo is located further away from the ideal position of the government parties (all government parties are partisan veto players in parliamentary systems). From the perspective of the veto player theory, greater distance between the veto players and the status quo increases the winset size. However, in contrast to the seat‐weighted alternation hypothesis, party size is not important in veto player theory, and veto players (parties) can block policy proposals outside of the winset irrespective of their relative size. According to veto player theory, if a seat‐weighted compromise lies outside of the winset (e.g., when the status quo is located at the position of, or close to the smaller party but further away from the bigger coalition partner, or lies in the core) it will not be approved. In this sense, the seat‐weighted compromise model is logically different and not overlapping with the veto player theory's predictions.

We note that the compromise model and the veto player theory allow us to test their predictive power by focusing on different aspects important for policy change. In the case of the compromise model we consider the distance between the status quo and the government position (demand for policy change). In the case of the veto player theory we consider the ideological distance between the veto players (obstacle for policy change). In this sense a direct comparison of the predictive power of the compromise model *against* the veto player theory on policy change as analysed in this work should be taken with a grain of salt. If one aspect (e.g., demand for policy change) outperforms the other (e.g., obstacles for policy change) in predicting policy change this should not be interpreted as a definite sign that one theoretical model (e.g., the compromise model) for policy making outperforms the other (veto player theory).^1^ Instead, the performance of the theoretical models should be considered and interpreted separately.

## Data and methods

### An original dataset on reform measures across 13 countries

To test the veto player prediction and the other hypotheses on policy change, we rely on original data on more than 5,600 important reform measures in the areas of social, taxation, labour and economic policy extracted from a systematic coding of country reports. For this purpose, we manually coded more than 1,000 periodical country reports issued on a quarterly basis by the EIU and the OECD on 13 Western European countries for a period of 20 years (c.1985–2005, covering the entire office periods of the cabinets beginning or ending closest to these dates). Our sample includes the following countries: Austria, Belgium, Denmark, Finland, France, Germany, Ireland, Italy, the Netherlands, Portugal, Spain, Sweden and the United Kingdom.

The EIU reports contain a large amount of information on recent economic and political developments and provide a regular review of the most pertinent policy‐making efforts and socioeconomic changes in a given country (EIU 1993, 2003). The leading goal of these reports is to provide reliable and comprehensive information about the current policy situation and concrete *policy changes* invoked by the government in a given country to potential business investors, international organisations, government agencies and academic institutes. EIU reports are prepared regularly by country experts with deep knowledge of the reform processes and policy developments in a country using various information channels, such as official statements, media reports and direct contact with government officials. According to interviews with former editors, country analysts follow common general guidelines for their reporting, the implementation of which is supervised by the central editors to ensure comparability in reporting across countries. To cross‐validate the EIU country reports’ coverage we also coded more than 200 country reports issued annually or biannually by the OECD. Whenever information on reform measures has been omitted in the EIU reports, but mentioned in the OECD Economic Surveys, we included these in our database.

To extract all reform measures mentioned in the EIU and OECD country reports we developed an extensive coding scheme which covers the four broad areas of social (welfare), economic, taxation and labour market policy. Research assistants were instructed to read a report, highlight reform measures and then enter the information into a database with around 50 variables. We code policy measures that change the policy status quo and were introduced, either by laws or decrees, through actions of the national government. Every *reform measure* is coded individually even if they occur in packages and we take into account only concrete measures which contain information on the policy instrument that is altered. For example, vague phrases such as ‘the government has started to fight rising levels of unemployment’ are not coded, as they do not refer to the specific action taken in order to reduce unemployment. In contrast, phrases such as ‘the budget proposal included a five‐day qualification period for receiving unemployment benefits’ (EIU 1993: 15) are coded as one reform measure.

The level of detail and style of reporting is best illustrated through a well‐known example of labour market and social policy reforms in Germany known as the “Hartz reforms”, introduced between 2002 and 2005 by the German SPD‐Greens government (e.g., Jacobi & Kluve 2006). These were implemented in four packages, comprising a large number of changes to the existing legal framework. In its January 2003 issue of the country report for Germany, the EIU published a concise summary of the political processes involved with the passage of the first package:

The government has started to implement the Hartz proposals, the recommendations made by a government commission headed by the Volkswagen personnel director Peter Hartz, presented in August 2002. … Those elements of the reform that require new legislation will be split between four bills. The first two laws have already been approved by the Bundestag, but on November 29th were vetoed by the Bundesrat. One of the two bills could still be implemented after the veto is overruled by a second vote in the Bundestag, because it does not require approval by the Bundesrat. The most important elements of this bill are as follows: … (EIU 2003: 25)

This is followed by a list and brief description of each important measure in the bill, such as: ‘The placement of unemployed people aged 55 years and older will also be facilitated by subsidies and an easing of employment protection rules for these people’ (EIU 2003: 25). Here, two reform measures were identified: one concerning the increase in job subsidies, and one capturing the deregulation of employment protection for older unemployed people.

**Figure 1:**
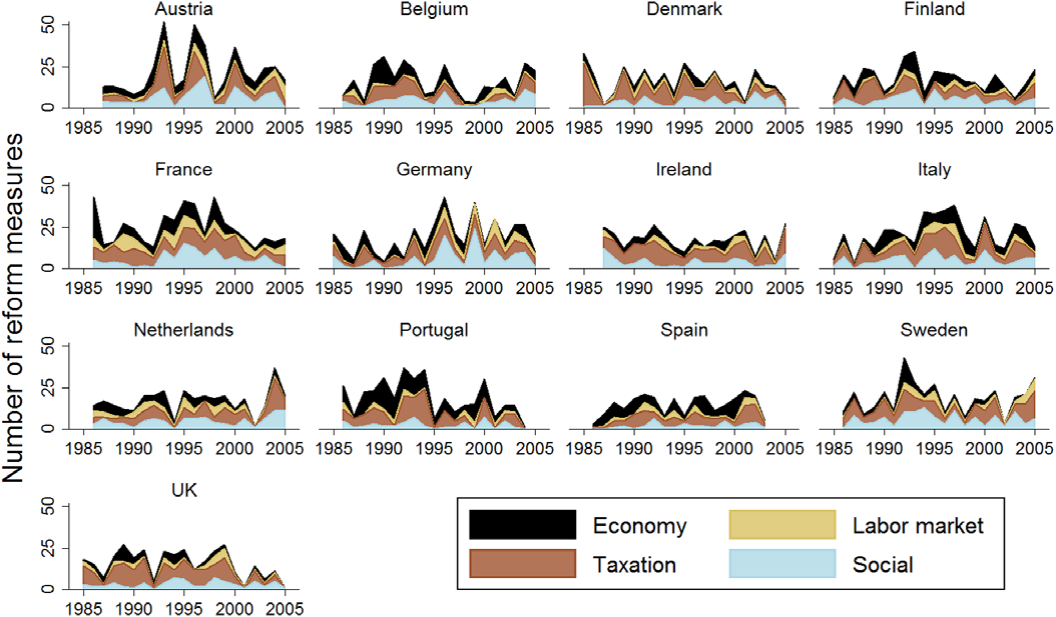
Number of reform measures by policy areas across countries and years. Notes: Area plots are stacked; therefore the peaks in each year show the total number of reform measures across all policy areas. Data is only shown between 1985 and 2005 to facilitate the graphical presentation (*n* = 260). [Color figure can be viewed at http://wileyonlinelibrary.com]

Figure 1 presents the number of reform measures, disaggregated into the four broad policy areas of our coding scheme. There is considerable variation in the number of reforms across countries, years and policy areas. In Austria, we see a considerable temporary slump in reform activity in 1994 and 1995, which is most likely due to parliament dissolution, general election and government formation in each year. The next peak in 2000 marks an example of a pronounced ideological alternation between cabinets, where the longstanding centrist coalition between the Social Democrats (SPÖ) and the Christian democratic People's Party (ÖVP) was replaced with a distinctly more right‐wing government of the People's Party and the Freedom Party (FPÖ). Another example of reform peaks, likely to be a result of ideological alternation between cabinets, can be found in Sweden, where the Social Democratic cabinet was replaced by the right‐of‐centre cabinet following the 1991 election. This cabinet introduced several new policies in an effort to combat the economic recession (Widfeldt 1995).

The reform output of governments thus seems to follow the legislative cycle and reflects institutional constraints and preference‐driven incentives (ideological alternation). Comparing the distribution of reforms across policy areas, we can see considerable within and cross‐country variation. In Germany, for example, the impact of the aforementioned Hartz reforms can be seen in the early 2000s: there is a marked rise in the number of labour market measures, which were previously less relevant on the reform agenda. In contrast to Germany, Austria and France, social policy reform measures are less prevalent in the United Kingdom, Ireland, Portugal and Spain.

### Methodological considerations

We show effect sizes and the predictive power of economic and political factors on governments’ reform output by analysing a count of the number of reform measures per country and year as our dependent variable. For each year, we count the total number of reform measures introduced by the cabinet which was the longest in office that year.

We analyse the number of reforms using a negative binomial model, an extension to the Poisson count model that allows the conditional variance to differ from the conditional mean (e.g., Long 1997). Negative binomial models are based on a maximum likelihood estimation. This allows us to estimate the models’ relative fit to the reform data based on log‐likelihood. We compare the Akaike information criterion (AIC; Akaike 1974), which penalises the improvement in the log‐likelihood for the number of additional parameters. Second, we calculate a ‘deviance‐based R^2^’ measure proposed by Cameron and Windmeijer (1997) as a goodness‐of‐fit measure for nonlinear regression models which is bounded between 0 and 1.^2^

Country‐level heterogeneity is a concern with most comparative data. A common solution is to estimate models with either fixed or random effects at the country level. Fixed effects models have the advantage that they control for time‐invariant factors within clusters. Since our variables of interest vary within countries, we estimate models with fixed effects using country dummies. This allows us to control for a multitude of country‐level factors that may explain differences in reform productivity, such as the electoral system, level of economic development or time‐invariant institutional factors such as powerful presidents or second chambers. We further calculate robust standard errors of the parameters based on these country clusters, as consecutive years within the same countries are most likely not to be independent from each other.^3^ A disadvantage of fixed effects is that they can absorb variance that is of substantive interest along with the time invariant factors (e.g., Plümper et al. 2005). We therefore run separate models with random intercepts on the country level and present their results in the Online Appendix.

### Independent variables

To evaluate the effect of economic crises on policy change (*H1*), we follow the approach by Wiese (2014) and construct two dummy variables indicating *recessions* and *unemployment crises*.^4^ First, we code a particular year as a recession whenever the growth in the annual gross domestic product (GDP) is negative. This definition is stricter than the common definition of recessions, which takes periods with two quarters of negative growth. Our measure therefore accounts for the requirement that crises must be sufficiently severe to induce reforms. Second, we operationalise episodes of unemployment crises whenever the annual unemployment rate is larger than 11.6 per cent (the sample mean plus one standard deviation). We use OECD data from the *Comparative Political Data Set* (Armingeon et al. 2015) to calculate these variables.

To evaluate the impact of seat‐weighted ideological alternation (*H2*) and ideological range (*H3*) we need measures of the positions of government parties. To take full advantage of the time‐variant structure of our data, we measure party positions using the *MARPOR (CMP)* coding of election manifestos (Volkens et al. 2015). We calculate the positions of each government party on a socioeconomic left–right dimension based on 16 categories using the logarithmic scaling suggested by Lowe et al. (2011) (see Table A1 in the Online Appendix for details).

To evaluate the alternation argument (*H2*), we calculate the distance between the incumbent cabinet in a given year and the policy status quo. We operationalise the position of the incumbent government with the seat‐weighted position of all cabinet parties. We approximate the position of the policy status quo via the average of the seat‐weighted position of all governments active four years prior to the current incumbent government. We therefore operationalise the status quo as the mean of yearly cabinet positions. For each cabinet at time *t*, we measure the status quo (SQ) as the mean of the government's position *G* over *k* number of years prior to the cabinet taking office:

(1) $$S\;Q_{t}=\frac{G_{t-1}+\ldots +G_{t-k}}{k}\;$$

We calculate the government position *G* in each year as the seat‐weighted average of the positions of all cabinet parties. For years where there were multiple cabinets in office, we choose the position of the cabinet that was the longest in office. We choose *k* to be four years, because this covers the duration of one full legislative term in many modern democracies. In addition, the four‐year time span introduces a time‐weight for the position of previous governments, where shorter governments contribute less to the location of the policy status quo.^5^ Our ideological alternation measure is then the absolute distance between the current government and the position of the status quo.

To test the effect of ideological range on policy change (*H3*), we calculate the ideological range between the most extreme veto players following Tsebelis (1999, 2002). We take the absolute distance between the veto player placed furthest to the left and the veto player placed furthest to the right for a given cabinet in a given year. We consider the ideological range between the veto players for the cabinet which has been in office the longest in each year. Our primary analysis incorporates only partisan veto players (all government parties), because the institutional veto players in our sample of countries can exercise their legislative veto only under restricted conditions, which are difficult to pinpoint for individual reform measures.^6^

We include three control variables to account for differences in reform output related to the legislative cycle and time available for policy making. Given that we count only the reform measures introduced by the longest‐lasting cabinet in a year, we include a variable that accounts for the fraction of days a government was in office per year. We include a dummy variable indicating whether a parliamentary election has taken place in a year. This controls for a possible slow‐down of reform activity related to general elections, when parties might be preoccupied with their electoral campaigns. We also control for the time left until the next scheduled election. We expect governments to be more active in the beginning of the legislative term, when coalition partners advance less controversial policy issues with a higher chance of swift adoption (Martin 2004). We also include cabinet type as a control variable, where 1 stands for a minimal winning cabinet and 0 for minority and oversized cabinets, as specified and measured in ParlGov (Döring & Manow 2016). Table 1 reports the descriptive statistics for all variables in our models.

**Table 1:** Descriptive statistics

	Mean	Standard deviation	Minimum	Median	Maximum
Number of reforms per year (DV)	17.66	9.17	1.00	18.00	52.00
Recession	0.08	0.28	0.00	0.00	1.00
Unemployment crisis	0.11	0.31	0.00	0.00	1.00
Seat‐weighted ideological alternation	0.89	0.77	0.00	0.71	3.80
Ideological range	1.11	1.14	0.00	0.97	4.55
Minimal winning cabinet (0/1)	0.53	0.50	0.00	1.00	1.00
Years left in legislative term (= days/365)	3.17	1.35	0.33	3.28	5.59
Election year (0/1)	0.28	0.45	0.00	0.00	1.00
Fraction of days in office per year	0.89	0.17	0.13	1.00	1.00

Note: *n* = 291.

## Empirical analysis

We present multivariate analyses predicting the number of reform measures introduced in a given country and a given year. In Table 2 we show the results from seven negative binomial regression analyses with fixed effects at the country level. Models 1, 2 and 3 estimate the effect of economic recession (*H1*), seat‐weighted ideological alternation (*H2*) and ideological range (*H3*), respectively. To gauge the relative predictive power of the models, we include different combinations of the factors in model 4 (economic recession and ideological alternation), model 5 (economic recession and ideological range) and model 6 (economic recession, ideological alternation and ideological range). Model 7 includes the same factors as model 6 with an interaction between ideological range and government type. The estimated coefficients are exponentiated to reflect incidence rate ratios. Values higher than 1 stand for a positive relationship and values smaller than 1 suggest a negative effect of the covariate. The dispersion parameter (α) is significantly larger than 0 in all models, which indicates that the count dependent variable is over‐dispersed and Poisson models are less appropriate than negative binomial regressions. We present the substantive effect sizes of our explanatory factors from models 6 and 7 by calculating average marginal effects in Table 3.

**Table 2:** Regression analysis of economic and social reform output in Western Europe

	Model 1	Model 2	Model 3	Model 4	Model 5	Model 6	Model 7
	Economy	Preferences	Veto player	Economy and Preferences	Economy and Veto player	Full	Interaction
*Economic demand*							
Recession	1.26** (0.09)			1.24** (0.09)	1.26*** (0.09)	1.25** (0.09)	1.21** (0.08)
Unemployment crisis	1.16 (0.11)			1.17 (0.12)	1.16 (0.11)	1.18 (0.12)	1.20* (0.11)
*Partisan policy demand*							
Seat‐weighted alternation		1.12** (0.04)		1.11** (0.04)		1.12*** (0.04)	1.11** (0.04)
*Veto players (VP)*							
Ideological range			1.01 (0.04)		0.99 (0.04)	0.98 (0.03)	1.03 (0.04)
MWC * Ideological range							0.88** (0.04)
*Control variables*							
Minimal winning cabinet (MWC)	1.05 (0.10)	1.10 (0.12)	1.04 (0.11)	1.11 (0.11)	1.05 (0.10)	1.12 (0.11)	1.30* (0.14)
Years left in legislative term	1.09*** (0.03)	1.10*** (0.03)	1.09*** (0.03)	1.09*** (0.03)	1.09*** (0.03)	1.09*** (0.03)	1.09*** (0.03)
Election year	0.83 (0.09)	0.81* (0.09)	0.84 (0.10)	0.80* (0.09)	0.83 (0.09)	0.80* (0.09)	0.80* (0.09)
Fraction of days in office/year	3.42*** (0.88)	3.18*** (0.82)	3.49*** (0.94)	3.13*** (0.80)	3.41*** (0.89)	3.11*** (0.81)	3.17*** (0.81)
Dispersion parameter (α)	0.15*** (0.02)	0.15*** (0.02)	0.15*** (0.02)	0.14*** (0.02)	0.15*** (0.02)	0.14*** (0.02)	0.14*** (0.02)
Observations	291	291	291	291	291	291	291
Deviance R^2^	0.284	0.284	0.266	0.301	0.284	0.302	0.313
Log‐likelihood	−993	−993	−997	−990	−993	−990	−987
AIC	2001	1999	2006	1995	2003	1997	1994

Notes: Exponentiated coefficients from negative binomial regressions with fixed effects at the country level and country‐clustered standard errors (*p < 0.05; **p < 0.01; ***p < 0.001). The dependent variable in all models is the number of reform measures introduced per country and year.

**Table 3:** Average marginal effects of covariates

Covariate	Model 6	Model 7
Ideological range	−0.33 [−1.38; 0.73]	–
Ideological range (minimal winning cabinets)	–	−1.74 [−2.69; −0.80]
Ideological range (Oversized and minority cabinets)	–	0.48 [−0.71; 1.67]
Seat‐weighted ideological alternation	1.95 [0.82; 3.08]	1.92 [0.71; 3.12]
Recession	3.99 [1.39; 6.58]	3.40 [1.07; 5.74]
Unemployment crisis	2.87 [−0.67; 6.41]	3.25 [0.10; 6.39]

Note: Average marginal effects with 95 per cent confidence intervals in brackets; calculated by holding all other variables at their observed values, except for the effect of ideological range in model 7, where the dummy variable for minimal winning coalitions was held at either 0 or 1.

Model 1 tests the impact of economic recession on policy change (*H1*) and includes the two dummy variables *recession* and *unemployment crisis*. The coefficients for both variables are positive, which means that governments introduce more reform measures in times of economic recessions. The incidence rate ratios reported in Table 2 reveal that governments introduce approximately 26 per cent more reform measures during years where the annual growth of the GDP is negative. Governments are also expected to introduce approximately 16 per cent more reform measures during years with a very high unemployment rate. However, the estimated standard errors of the unemployment coefficient are rather large and the estimated coefficient is not significant at conventional levels of significance.

**Figure 2:**
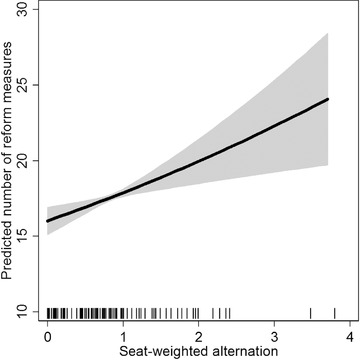
Effect of alternation on the predicted number of reform measures. Notes: Predictions are based on model 6. Other variables are held at their observed values. Grey area illustrates 95 per cent confidence intervals.

We test the effect of seat‐weighted ideological alternation on reform productivity (*H2*) in model 2. A one‐unit increase in the distance between the seat‐weighted left–right position of the incumbent government and the status quo increases the number of reform measures by about 12 per cent. This effect is illustrated in Figure 2, where we present the predicted number of reform measures for different values of ideological alternation (based on model 6 with all other variables). As the distance of the current government from the policy status quo increases from its minimum to its maximum, the predicted number of measures increases from around 18 to 25. This indicates that governments introduce more reform measures the more their policy platform differs from the status quo. This effect remains robust under the random effects specifications of the models and various other robustness tests (see the Online Appendix). We find a comparable predictive power of models 1 and 2 based on (measures derived from) the log‐likelihood of both models.

To evaluate the veto player hypothesis (*H3*) we start with model 3, which includes ideological range in addition to the control variables from models 1 and 2. The coefficient of ideological range in model 3 is virtually nil and positive, which is the opposite direction to what veto player theory predicts. Both the log‐likelihood and the deviance R^2^ are lower in model 3 compared to models 1 and 2, which indicates that ideological range (model 3) can explain a smaller share of the variation in reform productivity as compared to the individual models featuring economic recession (model 1) and ideological alternation (model 2).

**Figure 3:**
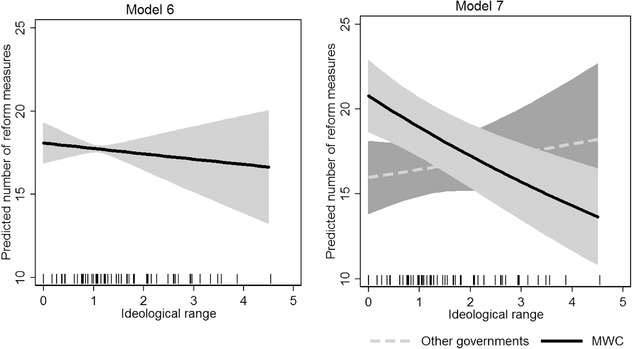
Effect of ideological range on the predicted number of reform measures. Notes: Predictions are based on model 7. Other variables are held at their observed values, except for the dummy variable for minimal winning coalitions, which was held at either 0 (minority and oversized governments) or 1 (minimal winning cabinets). Grey area illustrates 95 per cent confidence intervals.

We combine the covariates for economic crisis (*recession* and *unemployment crisis*), seat‐weighted alternation and ideological range in model 6. This model shows the effect of ideological range on policy change holding the level of economic recession and the ideological alternation between cabinets constant.^7^ The incidence rate ratio of ideological range here is smaller than 1, which reflects a negative effect of ideological range on policy change, in line with the veto player hypothesis. Nevertheless, the effect of ideological range remains statistically insignificant (see the left panel of Figure 3). The predictive power in model 6, which in addition to model 4 also includes ideological range, does not increase. The deviance R^2^ in both models remains practically the same (0.30). Further, the AIC value of 1997 for model 6 is higher than the AIC value of 1995 for model 4, which means that model 4 is more efficient and fits the data equally well with less parameters.

While we find that, on average, ideological range has no effect on policy change in these analyses, these results do not necessarily imply that the veto player theory has no predictive power *per se*. Rather, we draw attention to the definition of partisan veto players; in particular, that the agreement of partisan veto players is neither sufficient nor necessary for policy change. Which parties should ultimately be counted as veto players might differ across government types. All government parties should be sufficient and necessary to pass policy change in minimal winning governments. In contrast, at least one party in oversized coalitions is unnecessary for parliamentary majority formation, and is in this sense, not a veto player. In contrast, when minority governments rule, opposition parties can also have veto powers. Given these considerations and the fact that our operationalisation considers all government parties as veto players, ideological range should be a better predictor of policy change in minimal winning than in minority and oversized cabinets.

We address the question of whether the scope of the veto player hypothesis is limited to certain cabinet types in model 7, where we interact ideological range with a dummy for minimal winning cabinets. In this model, the effect of ideological range for minimal winning cabinets is statistically significant and substantively smaller than 1, indicating that greater ideological range results in a lower number of reform measures. Hence for minimal winning cabinets, a large ideological range between veto players results in greater policy stability.

We illustrate the magnitude of this conditional effect in the right panel of Figure 3, which depicts the number of predicted reform measures per year for different levels of ideological range and different cabinet types. The predicted number of reform measures per year decreases from around 21 to 14 as the ideological range between parties in minimal winning cabinets increases from its minimum to its maximum. The negative impact of ideological range on legislative production is less straightforward for other types of cabinets. In fact, ideological range for other types of cabinets has a marginally positive effect, albeit with large uncertainties in the estimates, as indicated by the 95 per cent confidence intervals.^8^

The effect size of the veto player prediction (ideological range) on reform productivity for minimal winning cabinets is comparable to the effect size of the seat‐weighted alternation (see model 7). An increase of ideological alternation from its 5^th^ to the 95^th^ percentile (i.e., from 0.03 to 2.28) results in an increase of about 4.4 reform measures per year based on model 7. Given a mean of about 17.7 reform measures per year, an increase of 4.4 reform measures constitutes about a 25 per cent increase in reform productivity – a substantively meaningful difference. Similarly, an increase in the ideological range from its 5^th^ to the 95^th^ percentile of its distribution (i.e., from 0 to 3.34) results in a predicted decrease of about 5.6 reform measures per year for minimal winning governments. Compared to a mean government productivity of 17.7 reform measures per year, a change from a low level of ideological range in cabinet (e.g., in one‐party governments) to a very high value (e.g., coalition government with high ideological conflict) means a predicted decrease in yearly reform productivity of about 32 per cent.

Model 7 considerably improves the predictive power compared to all other models. Model 7 has both the highest deviance R^2^ (about 0.31) as well as the lowest AIC value among all models at 1994. Also, the inclusion of the veto player variables and its interaction with government type considerably improves the overall explanatory power of the model as compared to model 4, which includes economic recession and alternation, and model 6, which includes all explanatory variables (economic recession, alternation and ideological range) but has no interaction between ideological range and government type.

## Concluding discussion

Reform making lies at the core of democratic governance. Failing to change the policy status quo when necessary may harm economic development, and lead to government and regime instability. Reform deadlock may lead to immobility and unstable political systems (Warwick 1992, 1994). Indecisiveness among veto players can further empower courts and bureaucrats with more freedom to interpret statutes and laws (Alivizatos 1995; Bednar et al. 1996).

Our study contributes to the literature on policy making by evaluating predictions on policy change drawn from the veto player theory. We do so using an original dataset with more than 5,600 important reform measures from four broad policy areas introduced in 13 Western European countries over a period of 20 years. One of the main virtues of the veto player theory is its parsimony. However, our findings suggest that the predictive power of the pure version of the veto player theory, which does not differentiate between different government types, is limited. We find a statistically significant negative effect of ideological range on the number of important government reform measures only for minimal winning governments.

These results have important implications for some of the theory's assumptions. Our findings call for a more precise definition of partisan veto players – in particular, a relaxation of the assumption that all government parties are partisan veto players (are necessary and sufficient for policy change) in parliamentary systems. For example, not all parties in oversized governments might have actual veto power, while governments with minority status rely on opposition parties to change the policy status quo. In such instances, veto player powers may depend upon various factors – for example, how much a party contributes to the parliamentary majority, their ideological placement in cabinet and parliament, their agenda‐setting powers (Rasch & Tsebelis 2011) and possibilities for parliamentary control. Accordingly, the identification of the actual partisan veto players may necessitate scholars looking into the black box of the policy‐making process in cabinet and parliament. Future research thus should aim at realising various synergies between existing theories of policy making, which emphasise the central role of prime ministerial powers, ministerial autonomy and various oversight and control mechanisms, with the veto player theory. Finally, research seeking to explain policy outcomes by differentiating between regime types may offer a partial explanation for our findings. As argued by Armingeon (2002) and Immergut and Abou‐Chadi (2014), ‘consociational’ democracies may be more efficient in introducing specific types of policies, such as measures reducing unemployment and inflation, despite governments being more inclusive. This rival explanation may be tested against veto player predictions by analysing the substantive content of reform measures.

We compared the predictive power of the veto player theory as the most encompassing theory focusing on political and institutional factors in reform politics with the predictive power of theoretical explanations drawn from the partisan and the political economy literatures. In line with partisan theory and coalition research, which highlights the importance of policy ‘compromise’, we find that ideological alternation – measured as the distance between the seat‐weighted position of a current government and the policy status quo – results in a greater number of reform measures. We also find strong support for the expectation that economic recessions drive policy changes. The effects of both features remain robust in the full model, making a considerable contribution to improving the model fit. However, we caution readers to interpret this as a ‘victory’ of either explanation over the veto player model. In particular, future tests of veto player theory against alternative political explanations should seek to develop a prediction for political conflict that allows a direct comparative test against the ‘ideological range hypothesis’ derived from the veto player theory. Alternatively, future research can modify the veto player theory so that it is possible to derive an equivalent of the ideological alternation measure from the perspective of the veto player theory and compare its performance against an alternation measure from other models for policy making.^9^

We further find that the legislative cycle plays a role in reform making, with governments introducing more reform measures in the beginning of the legislative term and considerably less during election years. These results connect to the literature on the political business and budget cycles, which suggests that politicians strategically time their policies according to the electoral cycle (Nordhaus 1975; Tufte 1978; Franzese 2002). Our results complement these analyses, which are typically based on macroeconomic developments, whereas we have information on the actual reform behaviour of governments. In future research, we suggest analysing reform making on a quarterly or monthly basis, allowing researchers to investigate the exact reasons for the negative effect of election years we found here.

Our findings highlight the importance of further theoretical developments and empirical tests of veto player theory as well as alternative political explanations for reform making. Future model building ought to involve the role of cabinet structure as well as various actors in parliament and government, such as the prime minister, ministers, coalition partners and opposition parties, in influencing policy outcomes. While Hallerberg and Hagen (1999) have already suggested that the party affiliation of the Prime Minister and Minister of Finance may undermine their capacity to act as wardens of a joint government policy, until recently little research has been focusing on the role of individual cabinet ministers and policy outputs in the area of economic policy making. Most previous studies have used some aggregate measure of partisanship within the cabinet to predict policy making. Important exceptions are the work by Becher (2010), Goodhart (2013), Herzog (2012) and Alexiadou (2013), who examine the impact of minister's background and their partisanship on specific policy outcomes. Martin and Vanberg (2014) suggest that coalition policies reflect a compromise between government parties rather than the preferences of the ministers proposing them. However, from the previous literature on coalition governance, we know that the institutional context matters to a great extent, suggesting that we need to evaluate such hypotheses using a larger sample of countries. Our data on reform measures in Western European countries allows scholars to do so.

## Supporting information


**Table A1. *MARPOR* categories used for socioeconomic left‐right dimension**.
**Table A2. GEE models with AR1 autocorrelation structure**.
**Table A3. Random effects models with varying intercepts at the country level**.
**Table A4. Fixed effects models with institutional veto player operationalization for ideological range**.
**Table A5. Fixed effects models analyzing the introduction of social, taxation and labor policy reform measures using a subsample of country/year observations**.

## Notes

1 Ideally, to compare the predictive powers for policy change of the compromise model against the veto player model we would need to focus on the same aspect or explanation for policy change (policy demand or government conflict) and derive observable implications or measures for these following the logic of the compromise model and the veto player theory. However, this would require a modification of either the veto player theory or the compromise model, which exceeds the goals of this article but presents an interesting avenue for future research. In particular, researchers who want to compare the effect of policy demand on policy change from the perspective of the compromise model and the veto player theory would need to measure the winset size (all policies which make all veto players better off) as defined by the veto player theory, which requires information about the location of the status quo. In contrast to the compromise model, the veto player theory cannot predict the exact location of the policy status quo without further information about the agenda‐setter, which is not specified within the veto player theory (Tsebelis 2002: 3). Therefore, to measure the winset size, researchers can extend the veto player theory by including existing assumptions about the agenda setter and its powers from the coalition policy making literature. Another possibility is to focus on government conflict (ideological range) as an explanation for policy change and policy stability and derive expectations about its impact from the perspective of the compromise model. Compared to the veto player theory, the coalition compromise model is not interested in explaining policy change *per se*, but rather in finding whose preferences prevail in coalition governments. Therefore, the compromise model does not provide a theoretical expectation that is as fleshed out and coherent regarding the effect of ideological distance between government parties on policy change. Future research might, however, focus on developing such expectations also from the perspective of the compromise model.

2 Both measures are calculated in Stata using the built‐in *estat ic* command and the module *devr2*. The formula for the deviance r‐squared is: DevianceR2=1−devianceoffittedmodel deviance  of  constant − only  model , whereas the AIC is calculated as: AIC=−2∗ln(likelihood)+2∗numberofparameters.

3 Given that we analyse time‐series cross‐sectional (TSCS) data, autocorrelation is another concern. In Table A2 in the Online Appendix, we show alternative models based on *generalised estimating equations* (GEE) that account both for potential autocorrelation as well as the count distribution of the dependent variable. Since the results from these models are very similar and the estimated autocorrelation within country clusters negligible, we present the results from conventional maximum likelihood models in the article.

4 We do not include changes in the inflation rate as a covariate since monetary policy is largely influenced by the central banks of individual countries until 1999 and the European Central Bank for the countries in the euro area after 1999. A robustness check shows that changes in the inflation rate do not alter the results presented below.

5 We obtain substantively similar results using other values for *k* number of years (e.g., the previous year or the previous three years, etc.) to define the policy status quo.

6 We run separate analyses where we consider both the partisan and institutional veto players in a given country and present our results in the Online Appendix. We find results in line with our main findings, with the difference being that the effect of ideological range for minimal winning cabinets is not significant at conventional levels.

7 Note that Tsebelis (1999) and Tsebelis and Chang (2004) also include economic and alternation variables in their statistical models testing the veto player theory. This makes model 6 more comparable to their results.

8 We show a number of robustness tests in the Online Appendix. We consider alternative model specifications (random effects), as well as include different operationalisations of our dependent variable and ideological range. In particular, we control for the possible influence of the EU on the policy agenda of national governments (West & Lee 2014) by excluding the economic policy area and five years after introduction of the Single European Act in 1987 from our sample. We find results similar to our main findings. We also run additional analyses where we measure ideological range by considering the positions of partisan *and* institutional veto players. We find results in line with our main findings, albeit with larger uncertainty estimates for the effect of ideological range among minimal winning cabinets.

9 See Note 1.
