# Supplementary material for: Veto player theory and reform making in Western Europe
Source: Eur J Polit Res. 2017 Jul 26;57(2):282–307. doi: 10.1111/1475-6765.12226 (PMC5900944; doi:10.1111/1475-6765.12226)
Supplement: Supplementary file 1 — Table A1. MARPOR categories used for socioeconomic left‐right dimension. Table A2. GEE models with AR1 autocorrelation structure. Table A3. Random effects models with varying intercepts at the country level. Table A4. Fixed effects models with institutional veto player operationalization for ideological range. Table A5. Fixed effects models analyzing the introduction of social, taxation and labor policy reform measures using a subsample of country/year observations. [file EJPR-57-282-s001.docx]

# Appendix: Veto Player Theory and Reform Making in Western Europe

This online appendix reports additional analyses and supporting information referred to in the main text. The dataset as well as the *Stata* do-file used for the analyses are made available on the website of the authors.^[[1]](#footnote-1)^

## *MARPOR categories for socioeconomic left-right position of parties*

Table A1 reports the *MARPOR* categories used to construct the socioeconomic left–right placements of parties on a one-dimensional scale (Volkens et al. 2015). These categories are used to calculate a logged left–right ratio based on the recommendations in Lowe et al. (2011).

**Table A1. *MARPOR* categories used for socioeconomic left-right dimension.**

| Left | | Right | |
| --- | --- | --- | --- |
| per403 | Market Regulation: Positive | per401 | Free Market Economy |
| per404 | Economic Planning: Positive | per402 | Incentives: Positive |
| per406 | Protectionism: Positive | per407 | Protectionism: Negative |
| per409 | Keynesian Demand Management | per414 | Economic Orthodoxy |
| per415 | Marxist Analysis | per505 | Welfare State Limitation |
| per412 | Controlled Economy: Positive | per702 | Labour Groups: Negative |
| per413 | Nationalisation: Positive |  |  |
| per503 | Equality: Positive |  |  |
| per504 | Welfare State Expansion: Positive |  |  |
| per701 | Labour Groups: Positive |  |  |

## *Alternative model specifications: GEE models with AR1 autocorrelation*

We here present alternative specifications for the models presented in the main text as robustness tests. First, we control for potential autocorrelation in our dependent variable by estimating *Generalized Estimating Equations* (*GEE*; see Zorn 2001) in Table A2. These models are estimated using the *xtgee* command in Stata, assuming a negative binomial distribution of the dependent variable and an AR1 autocorrelation structure within countries. Estimates of the dispersion parameters (*alpha*) were obtained from maximum likelihood (ML) models with the same covariates. The number of observations (country/years) differs from the other analyses since the AR1 autocorrelation structure necessitates that there are no gaps in the time-series. There is one year missing in the Italian case due to the lack of positional data for the technocratic Dini cabinet, which excludes Italy from the sample. Nevertheless, the estimated coefficients, which are exponentiated and can be interpreted like those from the ML negative binomial models, are very similar to the coefficients presented in Table 2 in the main text. Seat-weighted alternation and economic recessions have a positive effect on the number of reform measures, while the effect of ideological range is only negative among minimal winning cabinets. Specifically, the average marginal effect (*AME*; see Table 3 in main text for comparison) of the ideological range variable is -1.67 (CI[-2.60; -0.73]) and thus substantially negative among minimal winning cabinets, while it is marginally positive and not significant for other cabinet types (0.29, CI[-1.27; 1.80]). The estimated *rho* parameter in the GEE models further suggest that the AR1 autocorrelation in our dependent variable is fairly small.

**Table A2. GEE models with AR1 autocorrelation structure.**

|  | Model 1  Economy | Model 2  Preferences | Model 3  VP | Model 4  Economy & Preferences | Model 5  Economy & VP | Model 6  Full | Model 7  Interaction |
| --- | --- | --- | --- | --- | --- | --- | --- |
| *Economic demand* |  |  |  |  |  |  |  |
| Recession | 1.26^***^ |  |  | 1.25^**^ | 1.27^***^ | 1.27^***^ | 1.24^**^ |
|  | (0.08) |  |  | (0.09) | (0.08) | (0.09) | (0.08) |
|  |  |  |  |  |  |  |  |
| Unemployment crisis | 1.17 |  |  | 1.19 | 1.18 | 1.20 | 1.21^*^ |
|  | (0.11) |  |  | (0.11) | (0.11) | (0.11) | (0.10) |
|  |  |  |  |  |  |  |  |
| *Partisan policy demand* |  |  |  |  |  |  |  |
| Seat-weighted alternation |  | 1.13^**^ |  | 1.12^***^ |  | 1.13^***^ | 1.12^***^ |
|  |  | (0.04) |  | (0.04) |  | (0.03) | (0.04) |
|  |  |  |  |  |  |  |  |
| *Veto players* |  |  |  |  |  |  |  |
| Ideological range |  |  | 1.01 |  | 0.98 | 0.97 | 1.02 |
|  |  |  | (0.05) |  | (0.05) | (0.03) | (0.05) |
|  |  |  |  |  |  |  |  |
| MWC * Ideological range |  |  |  |  |  |  | 0.90 |
|  |  |  |  |  |  |  | (0.05) |
|  |  |  |  |  |  |  |  |
| *Control variables* |  |  |  |  |  |  |  |
| Minimal winning cabinet | 1.08 | 1.13 | 1.06 | 1.15 | 1.08 | 1.15 | 1.31^*^ |
|  | (0.11) | (0.13) | (0.12) | (0.11) | (0.11) | (0.11) | (0.16) |
|  |  |  |  |  |  |  |  |
| Years left in legislative term | 1.09^***^ | 1.10^***^ | 1.10^***^ | 1.09^***^ | 1.09^***^ | 1.09^***^ | 1.09^***^ |
|  | (0.03) | (0.03) | (0.03) | (0.03) | (0.03) | (0.03) | (0.03) |
|  |  |  |  |  |  |  |  |
| Election year | 0.79^*^ | 0.78^*^ | 0.80 | 0.76^**^ | 0.79^*^ | 0.76^**^ | 0.76^**^ |
|  | (0.09) | (0.08) | (0.09) | (0.08) | (0.08) | (0.08) | (0.08) |
|  |  |  |  |  |  |  |  |
| Fraction of year in office | 3.26^***^ | 3.12^***^ | 3.37^***^ | 3.01^***^ | 3.27^***^ | 3.01^***^ | 3.01^***^ |
|  | (0.87) | (0.88) | (0.93) | (0.81) | (0.86) | (0.82) | (0.80) |
|  |  |  |  |  |  |  |  |
| Dispersion parameter (α) | 0.137 | 0.138 | 0.144 | 0.131 | 0.137 | 0.130 | 0.127 |
| Estimated rho (AR1) | 0.117 | 0.143 | 0.144 | 0.118 | 0.120 | 0.122 | 0.104 |
| Observations | 269 | 269 | 269 | 269 | 269 | 269 | 269 |

*Note:* Exponentiated coefficients with country-clustered standard errors in parentheses (^*^ *p* < 0.05, ^**^ *p* < 0.01, ^***^ *p* < 0.001); coefficients for country fixed effects and intercepts not reported.

## *Alternative model specifications: Random effects models*

In the methodology section, we have briefly addressed the criticism of fixed effects in comparison with random effects model specifications. While, for instance, fixed effects control for time-invariant factors within clusters, they may absorb variance that is of substantive interest as well (e.g., Plümper et al. 2005). On the other hand, there has been some recent methodological debate whether random effects models are appropriate when the number of clusters (here: countries) is small (Bryan & Jenkins 2016). As this is the case with our sample of 13 countries, we have focused on the fixed effects specification in the manuscript. Country-level heterogeneity is handled differently in a random intercept model than in a fixed effects model (see Gelman & Hill 2007). In our case, this concerns the time-invariant differences between countries, which are absorbed by the dummy variables in the fixed effects model. The estimates from the random intercept models, on the other hand, can also reflect differences between units (hence also called ‘partial pooling’ models). In Table A3 we thus present our analyses based on random effects models with varying intercepts.

**Table A3. Random effects models with varying intercepts at the country level.**

|  | Model 1  Economy | Model 2  Preferences | Model 3  VP | Model 4  Economy & Preferences | Model 5  Economy & VP | Model 6  Full | Model 7  Interaction |
| --- | --- | --- | --- | --- | --- | --- | --- |
| *Economic demand* |  |  |  |  |  |  |  |
| Recession | 1.25^*^ |  |  | 1.23^*^ | 1.23^*^ | 1.22^*^ | 1.20 |
|  | (0.12) |  |  | (0.12) | (0.12) | (0.12) | (0.12) |
|  |  |  |  |  |  |  |  |
| Unemployment crisis | 1.00 |  |  | 1.03 | 1.00 | 1.03 | 1.05 |
|  | (0.11) |  |  | (0.11) | (0.11) | (0.11) | (0.12) |
|  |  |  |  |  |  |  |  |
| *Partisan policy demand* |  |  |  |  |  |  |  |
| Seat-weighted alternation |  | 1.10^*^ |  | 1.10^*^ |  | 1.10^*^ | 1.10^*^ |
|  |  | (0.04) |  | (0.04) |  | (0.04) | (0.04) |
|  |  |  |  |  |  |  |  |
| *Veto players* |  |  |  |  |  |  |  |
| Ideological range |  |  | 1.03 |  | 1.01 | 1.01 | 1.04 |
|  |  |  | (0.03) |  | (0.03) | (0.03) | (0.04) |
|  |  |  |  |  |  |  |  |
| MWC * Ideological range |  |  |  |  |  |  | 0.93 |
|  |  |  |  |  |  |  | (0.05) |
|  |  |  |  |  |  |  |  |
| *Control variables* |  |  |  |  |  |  |  |
| Minimal winning cabinet | 1.02 | 1.06 | 1.03 | 1.06 | 1.02 | 1.06 | 1.16 |
|  | (0.07) | (0.07) | (0.07) | (0.07) | (0.07) | (0.07) | (0.12) |
|  |  |  |  |  |  |  |  |
| Years left in legislative term | 1.10^***^ | 1.10^***^ | 1.10^***^ | 1.10^***^ | 1.10^***^ | 1.10^***^ | 1.10^***^ |
|  | (0.03) | (0.03) | (0.03) | (0.03) | (0.03) | (0.03) | (0.03) |
|  |  |  |  |  |  |  |  |
| Election year | 0.83^*^ | 0.80^*^ | 0.83^*^ | 0.80^*^ | 0.83^*^ | 0.80^*^ | 0.80^*^ |
|  | (0.07) | (0.07) | (0.07) | (0.07) | (0.07) | (0.07) | (0.07) |
|  |  |  |  |  |  |  |  |
| Fraction of year in office | 3.24^***^ | 2.97^***^ | 3.26^***^ | 2.98^***^ | 3.25^***^ | 2.99^***^ | 3.05^***^ |
|  | (0.80) | (0.74) | (0.81) | (0.73) | (0.80) | (0.74) | (0.75) |
|  |  |  |  |  |  |  |  |
| Country-level variance | 0.01 | 0.01 | 0.01 | 0.01 | 0.01 | 0.01 | 0.02 |
|  | (0.01) | (0.01) | (0.01) | (0.01) | (0.01) | (0.01) | (0.01) |
| Observations | 291 | 291 | 291 | 291 | 291 | 291 | 291 |
| Log likelihood | -1005 | -1005 | -1007 | -1002 | -1005 | -1002 | -1002 |
| AIC | 2028 | 2025 | 2031 | 2025 | 2030 | 2027 | 2027 |

*Note:* Exponentiated coefficients from random intercept models with standard errors in parentheses (^*^ *p* < 0.05, ^**^ *p* < 0.01, ^***^ *p* < 0.001); coefficients for intercepts not reported.

The main differences for our results is that the random effects specification indicates a smaller effect of ideological range for minimal winning cabinets. Based on Model 7 in Table A3, the average marginal effect of ideological range among minimal winning cabinets is merely -0.61 (CI[-2.32; 1.09]) and 0.60 (CI[-0.66; 1.85]) for other government types (see right column of Table 3 in the main text for comparison). Thus, while changes in the ideological range between minimal winning cabinets may explain differences in reform productivity within a country, the flat slope here casts doubt on whether the ideological distance between government parties can explain differences in reform activity across countries. The substantive interpretation of the effects of other covariates remains comparable to the fixed effects models.

## *Considering institutional veto players in the operationalization of ideological range*

Our primary analysis further only considered partisan veto players. Following Tsebelis (1999, 2004), we run separate analyses where, in addition to the government parties, we also consider the institutional veto players present in some countries.

All variables in the models in Table A4 are thus identical to the models presented in Table 2 in the main text except for ideological range. The ideological range of a cabinet here is calculated by treating institutional veto players as additional veto players whenever they are distinct from the cabinet parties. This concerns the party of the president in France, who has veto powers only over government decrees, the party of the president in Portugal (Amorim Neto 2003), as well as the majority parties in the German *Bundestag* (second chamber), which can veto so-called approval laws (Zustimmungsgesetze) constituting approximately 53 percent of the overall German legislation. For Portugal, we add a veto player to the government whenever the president was from a different party than the government, which was the case from 1985–1995. Similarly, we account for the party of the French president during periods of ‘cohabitation’. For Germany, we code opposition parties as veto players during years where opposition parties held the majority of seats in the Bundesrat. Since we cannot control over which reform cases these institutional veto players actually had veto powers, we follow Tsebelis and Chang (2004) and make the assumption that they had a veto over all reform measures.

The measures for ideological range thus incorporate the left–right positions of the presidents in France and Portugal as well as the positions of the opposition parties in the second chamber in Germany when these controlled a majority in the Bundesrat. Based on this operationalization, ideological range has a smaller negative effect on reform productivity among minimal winning cabinets, which is insignificant at conventional levels. Specifically, the average marginal effect of ideological range among these cabinets is -0.76 (CI [-1.87; 0.34]) and 0.76 (CI [-0.55; 2.07]) for oversized and minority cabinets. Note that, in these analyses we follow Tsebelis’s (1999: 601) operationalization for government types, where minimal winning cabinets that require the support of opposition parties in the second chamber are considered ‘oversized’. We urge the reader to interpret these results with caution. First, the operationalization of oversized governments used by Tsebelis (1999: 601) goes against the common classification of these governments. Second, the institutional veto players in our sample of countries do not have veto rights concerning all relevant decisions, which might explain the weaker findings when partisan and institutional veto players are taken into account.

**Table A4. Fixed effects models with institutional veto player operationalization for ideological range.**

|  | Model 1  Economy | Model 2  Preferences | Model 3  VP | Model 4  Economy & Preferences | Model 5  Economy & VP | Model 6  Full | Model 7  Interaction |
| --- | --- | --- | --- | --- | --- | --- | --- |
| *Economic demand* |  |  |  |  |  |  |  |
| Recession | 1.26^***^ |  |  | 1.25^***^ | 1.24^***^ | 1.24^***^ | 1.22^***^ |
|  | (0.08) |  |  | (0.08) | (0.07) | (0.07) | (0.06) |
|  |  |  |  |  |  |  |  |
| Unemployment crisis | 1.13 |  |  | 1.14 | 1.13 | 1.14 | 1.17 |
|  | (0.12) |  |  | (0.12) | (0.12) | (0.12) | (0.12) |
|  |  |  |  |  |  |  |  |
| *Partisan policy demand* |  |  |  |  |  |  |  |
| Seat-weighted alternation |  | 1.10^*^ |  | 1.10^**^ |  | 1.10^**^ | 1.10^*^ |
|  |  | (0.04) |  | (0.04) |  | (0.04) | (0.04) |
|  |  |  |  |  |  |  |  |
| *Veto players* |  |  |  |  |  |  |  |
| Ideological range |  |  | 1.05 |  | 1.03 | 1.02 | 1.04 |
|  |  |  | (0.04) |  | (0.04) | (0.04) | (0.04) |
|  |  |  |  |  |  |  |  |
| MWC * Ideological range |  |  |  |  |  |  | 0.92 |
|  |  |  |  |  |  |  | (0.04) |
|  |  |  |  |  |  |  |  |
| *Control variables* |  |  |  |  |  |  |  |
| Minimal winning cabinet | 0.98 | 0.99 | 0.98 | 1.01 | 1.00 | 1.02 | 1.16 |
|  | (0.08) | (0.08) | (0.09) | (0.08) | (0.09) | (0.08) | (0.14) |
|  |  |  |  |  |  |  |  |
| Years left in legislative term | 1.09^***^ | 1.10^***^ | 1.09^***^ | 1.09^***^ | 1.09^***^ | 1.09^***^ | 1.09^***^ |
|  | (0.03) | (0.03) | (0.03) | (0.03) | (0.03) | (0.03) | (0.03) |
|  |  |  |  |  |  |  |  |
| Election year | 0.83 | 0.81 | 0.84 | 0.81^*^ | 0.83 | 0.81 | 0.81 |
|  | (0.09) | (0.09) | (0.09) | (0.09) | (0.09) | (0.09) | (0.09) |
|  |  |  |  |  |  |  |  |
| Fraction of year in office | 3.41^***^ | 3.19^***^ | 3.42^***^ | 3.15^***^ | 3.39^***^ | 3.15^***^ | 3.20^***^ |
|  | (0.88) | (0.81) | (0.91) | (0.80) | (0.88) | (0.81) | (0.82) |
|  |  |  |  |  |  |  |  |
| Dispersion parameter | 0.15^***^ | 0.15^***^ | 0.15^***^ | 0.14^***^ | 0.14^***^ | 0.14^***^ | 0.14^***^ |
|  | (0.02) | (0.02) | (0.02) | (0.02) | (0.02) | (0.02) | (0.02) |
| Observations | 291 | 291 | 291 | 291 | 291 | 291 | 291 |
| Log likelihood | -994 | -994 | -996 | -991 | -993 | -990 | -989 |
| AIC | 2001 | 2000 | 2004 | 1997 | 2002 | 1999 | 1998 |

*Note:* Exponentiated coefficients from negative binomial regressions; country-clustered standard errors in parentheses (^*^ *p* < 0.05, ^**^ *p* < 0.01, ^***^ *p* < 0.001); coefficients for country fixed effects and intercepts not reported.

## *Considering the influence of EU legislation on reform output*

Lastly, we check for the robustness of our analyses by considering the importance of EU legislation for policy making on the national level. As we have collected information not only on the presence but also on the content of reform measures, we can test the robustness of our results by looking at a subset of policy areas where there is less influence of EU legislation on the policy making of national governments. In the case of our four policy areas social, taxation, labor and economy, the latter is by far the one policy area where the EU has the strongest presence in national legislation. An analysis by Angelova et al. (2012) shows that 30.9 per cent of EU directives are related specifically to the internal market. Yet labor and social policy make up only 5.2 per cent of EU directives combined. We thus have good reason to believe that national policy making in the latter two policy areas is influenced to a lesser extent by EU legislation than economic policy measures. Second, we consider the importance of the Single European Act for all countries that were in the EC at the time, starting from its introduction in 1987 until 1992, which marks the planned date to finalize the creation of the single market (see e.g., West and Lee 2014). Our robustness tests are presented in Table A4.

We here seek to minimize the concerns for potential contamination of our data by EU legislation in two ways: first, we exclude economic policy measures entirely from the coding of our dependent variable and count only social, taxation and labor measures introduced in a given country/year. Second, for countries that were members of the EC when the Single European Act came into effect in 1987, we drop all observations up until and including the year 1992 from our sample. Consequently, the number of observations (*n* = 231) as well as the number of reform measures considered as the dependent variable in Table A4 is lower than in the main analysis (*mean =* 13.05; *SD =* 7.54; *median =* 12). Despite this rather drastic subsampling, the results do not change substantively. All estimated coefficients have the same sign, as well as similar sizes and uncertainties as in Table 2 in the main text. We thus believe that our findings offer robust insights into the factors influencing reform making on the national level and are not driven by EU legislation.

**Table A5. Fixed effects models analyzing the introduction of social, taxation and labor policy reform measures using a subsample of country/year observations.**

|  | Model 1  Economy | Model 2  Preferences | Model 3  VP | Model 4  Economy & Preferences | Model 5  Economy & VP | Model 6  Full | Model 7  Interaction |
| --- | --- | --- | --- | --- | --- | --- | --- |
| *Economic demand* |  |  |  |  |  |  |  |
| Recession | 1.22^*^ |  |  | 1.18 | 1.21^*^ | 1.18 | 1.11 |
|  | (0.11) |  |  | (0.12) | (0.12) | (0.14) | (0.12) |
|  |  |  |  |  |  |  |  |
| Unemployment crisis | 0.88 |  |  | 0.87 | 0.88 | 0.87 | 0.88 |
|  | (0.16) |  |  | (0.17) | (0.16) | (0.18) | (0.16) |
|  |  |  |  |  |  |  |  |
| *Partisan policy demand* |  |  |  |  |  |  |  |
| Seat-weighted alternation |  | 1.15^**^ |  | 1.14^**^ |  | 1.14^**^ | 1.14^**^ |
|  |  | (0.06) |  | (0.05) |  | (0.05) | (0.06) |
|  |  |  |  |  |  |  |  |
| *Veto players* |  |  |  |  |  |  |  |
| Ideological range |  |  | 1.02 |  | 1.00 | 1.00 | 1.07 |
|  |  |  | (0.05) |  | (0.05) | (0.05) | (0.07) |
|  |  |  |  |  |  |  |  |
| MWC * Ideological range |  |  |  |  |  |  | 0.87^*^ |
|  |  |  |  |  |  |  | (0.06) |
|  |  |  |  |  |  |  |  |
| *Control variables* |  |  |  |  |  |  |  |
| Minimal winning cabinet | 1.04 | 1.11 | 1.06 | 1.08 | 1.04 | 1.08 | 1.32^*^ |
|  | (0.15) | (0.15) | (0.16) | (0.14) | (0.15) | (0.15) | (0.18) |
|  |  |  |  |  |  |  |  |
| Years left in legislative term | 1.12^***^ | 1.12^***^ | 1.12^***^ | 1.13^***^ | 1.12^***^ | 1.13^***^ | 1.12^***^ |
|  | (0.03) | (0.03) | (0.03) | (0.03) | (0.03) | (0.03) | (0.03) |
|  |  |  |  |  |  |  |  |
| Election year | 0.71^**^ | 0.67^***^ | 0.71^**^ | 0.67^***^ | 0.71^**^ | 0.67^***^ | 0.67^**^ |
|  | (0.09) | (0.08) | (0.09) | (0.08) | (0.09) | (0.08) | (0.08) |
|  |  |  |  |  |  |  |  |
| Fraction of year in office | 2.50^**^ | 2.16^*^ | 2.49^**^ | 2.19^*^ | 2.50^**^ | 2.19^*^ | 2.27^*^ |
|  | (0.87) | (0.73) | (0.88) | (0.73) | (0.87) | (0.73) | (0.78) |
|  |  |  |  |  |  |  |  |
| Dispersion parameter (α) | 0.18^***^ | 0.17^***^ | 0.18^***^ | 0.17^***^ | 0.18^***^ | 0.17^***^ | 0.16^***^ |
|  | (0.03) | (0.03) | (0.03) | (0.03) | (0.03) | (0.03) | (0.03) |
| Observations | 231 | 231 | 231 | 231 | 231 | 231 | 231 |
| Log likelihood | -745 | -742 | -746 | -741 | -745 | -741 | -739 |
| AIC | 1503 | 1497 | 1504 | 1498 | 1505 | 1500 | 1498 |

*Note:* Exponentiated coefficients from negative binomial regressions; country-clustered standard errors in parentheses (^*^ *p* < 0.05, ^**^ *p* < 0.01, ^***^ *p* < 0.001); coefficients for country fixed effects and intercepts not reported.

## *References*

Angelova, M., Dannwolf, T. & König, T. (2012). How robust are compliance findings? A research synthesis. *Journal of European Public Policy*, 19(8): 1269–1291.

Amorim Neto, O. (2003). Portugal: Changing patterns of delegation and accountability under the President’s watchful eyes, in K. Strøm, W.C. Müller & T. Bergman (eds.), *Delegation and accountability in parliamentary democracies*. Oxford: Oxford University Press, 552–572.

Bryan, M. L. & Jenkins, S. (2016). Multilevel modelling of country effects: A cautionary tale, *European Sociological Review* 32(1): 3-22.

Gelman, A. & Hill, J. (2007). *Data analysis using regression and multilevel/hierarchical models*. Cambridge: Cambridge University Press.

Plümper, T., Troeger, V. E. & Manow, P. (2005). Panel data analysis in comparative politics: Linking method to theory. *European Journal of Political Research* 44(2): 327–354.

Tsebelis, G. (1999). Veto players and law production in parliamentary democracies: An empirical analysis. *American Political Science Review* 93(3): 591–608.

Tsebelis, G. & Chang, E. C. (2004). Veto players and the structure of budgets in advanced industrialized countries. *European Journal of Political Research* 43(3): 449–476.

West, K. J. & Lee, H. (2014). Veto players revisited: Internal and external factors influencing policy production. *Legislative Studies Quarterly* 39(2): 227–260.

Zorn, C. J. W. (2001). Generalized estimating equation models for correlated data: A review with applications. *American Journal of Political Science* 45(2): 470–490.

1. <http://staatswissenschaft.univie.ac.at/en/team/head-of-department/wolfgang-c-mueller/> [↑](#footnote-ref-1)
